# A cost-of-illness study of Behçet syndrome in Italy

**DOI:** 10.1007/s10198-023-01593-8

**Published:** 2023-05-22

**Authors:** Valentina Lorenzoni, Diana Marinello, Ilaria Palla, Marta Mosca, Giuseppe Turchetti, Rosaria Talarico

**Affiliations:** 1 Institute of Management, Scuola Superiore Sant’Anna, Piazza Martiri Della Libertà n. 33, 56127 Pisa, Italy; 2 Rheumatology Unit, Azienda Ospedaliero Universitaria Pisana, University of Pisa, Pisa, Italy

**Keywords:** Behçet, Cost-of-illness, Economic, I12, I18

## Abstract

**Objective:**

This study aims at evaluating the cost-of-illness (COI) of patients diagnosed with Behcet’s syndrome (BS) in Italy, trying to depict the impact of different costs’ components to the overall economic burden and analysing the variability of costs according to years since diagnosis and age at first symptoms.

**Methods:**

With a cross-sectional evaluation, we surveyed a large sample of BS patients in Italy assessing several dimensions related to BS, also including fact related to the use of health resources utilization, formal and informal care, and productivity losses. Overall costs, direct health, direct non-health, and indirect costs were thus estimated per patient/year considering a Societal perspective and the impact of years since diagnosis, age at first symptoms on costs was evaluated using generalized linear model (GLM) and a two-part model, adjusting for age and distinguishing among employed and non-employed responders.

**Results:**

A total of 207 patients were considered in the present study. From the perspective of the Society, mean overall costs for BS patient were estimated to be 21,624 € (0;193,617) per patient/year. Direct non-health expenses were the main costs component accounting for 58% of the overall costs, followed direct health costs, 36%, while indirect costs because of productivity losses represented 6% of the overall costs. Being employed resulted in significantly lower overall costs (*p* = 0.006). Results from the multivariate regression analyses suggested that the probability of incurring in overall costs equal to zero decreased as time from BS diagnosis is 1 year or more as compared to newly diagnosed patients (*p* < 0.001); while among those incurring in expenses, costs decreased for those experiencing first symptoms between 21 and 30 years (*p* = 0.027) or later (*p* = 0.032) as compared to those having symptoms earlier. Similar findings emerged among the subgroups of patients declaring themselves as workers, while no impact of years since diagnosis or age of first symptoms was found among non-workers.

**Conclusions:**

The present study offers a comprehensive overview of the economic consequences imposed by BS in a societal perspective, providing insights into the distribution of the different costs component related to BS, thus helping the development of targeted policies.

**Supplementary Information:**

The online version contains supplementary material available at 10.1007/s10198-023-01593-8.

## Introduction

Despite the rarity of the single disease, overall rare diseases (RDs) are estimated to affect 300 millions of people worldwide [1] with also a considerable economic burden.

Understanding the economic burden of RDs is of paramount importance to assess the potential value of technological innovations (either drugs or device) and to establish priorities to develop policies aiming at supporting patients and their family. The possibility of containing health costs for national health systems (NHS) and the society follows thereafter.

Nevertheless, at present, existing studies focusing on the cost of illness (COI) of RDs are scarce [2].

Behcet’s syndrome (BS) is a rare multisystem vasculitis primarily characterized by recurrent urogenital ulcers and sight threatening uveitis [3], and even other involvement could be present such as joints, skin, vessels, central nervous system (CNS), and gastrointestinal system [4, 5]. The complexity of the BS phenotype variability requires a multi-disciplinary view and of course a personalised approach to the individual patient to improve the overall disease prognosis and the quality of life of both the patient and the caregivers/family members [6].

To our knowledge, there are very few studies exploring the economic dimension of BS [7] and there is a paucity of information about the cost of the disease in a contemporary European population, nor information on the impact of different cost components to the overall economic burden, nor evidence about the variability of costs according to disease duration and patients’ characteristics.

Therefore, the aim of the present study is to estimate the cost-of-illness of patients diagnosed with BS in Italy. In particular, an ad hoc survey was conducted on a large sample of BS patients in Italy to assess several dimensions, including data regarding direct and indirect costs related to BS.

More specifically, we aimed at quantifying the direct and indirect costs of BS in a large sample of Italian patients, understanding the impact of different costs components to the overall economic burden and at analysing the variability of these costs according to years since diagnosis and age at first symptoms. Moreover, given both the relatively high proportion of patients declaring themselves to be not employed, like retired people, students, housewives and economically unemployed, and the impact of working condition on costs (i.e., in term of indirect costs), analyses were also conducted distinguishing between employed and not employed.

## Methods

### Study design and population

A cross-sectional study was conducted to investigate several dimensions related to BS, including the use of resources related to direct and indirect costs. In details, an ad hoc questionnaire was co-designed by clinicians’ expert in the management of BS, health economists, patients’ representative, and caregivers in collaboration with the Italian Association for Behçet Disease (SIMBA OdV) [8]. An English version of the questionnaire used in the study is reported as Online resource 1. The questionnaire was implemented online using EU Survey [9] and promoted among Italian BS patients trough different dissemination channels with the support of SIMBA OdV that contributed to the dissemination of the survey (i.e., website, Social media, etc.). The survey was accessible online for 3 months, from the 5th of July 2019 to the 5th October 2019. Participation to the questionnaire was voluntary and data were collected anonymously, a clear statement of consent was filled by the patients that responded to the questionnaire, and therefore, the institutional review board (IRB) was not requested.

### Collection of data related to resource use and cost estimation

Among other information, the questionnaire also included specific questions aiming at assessing resource use related to direct and indirect costs over 6 months preceding the date of assessment.

In detail, resources use related to hospital admissions, emergency department (ED) visits, outpatient care (specialists visits, laboratory exams and imaging evaluations), drugs and out-of-pocket expenses comprising the patients’ additional payments for prescribed treatments, and other disease-related expense were collected to derive direct health costs. Moreover, hours of formal and informal assistance, costs for travels and accommodations because of BS-related consultations and working days lost were collected to estimate, respectively, direct non-health costs and indirect costs for productivity loss.

Cost assessment was performed by applying the perspective of the Society.

To estimate direct health costs resources related to hospital admission, ED visits, and outpatient care were valued according to the national price lists [10]. The details of unit costs considered to value those resources are reported in Table S1. The costs related to prescribed drugs were estimated considering drug dosage, frequency of use collected from the questionnaire, and national list prices [11, 12].

The amount of out-of-pocket expenses related to patients’ costs for dispensed drugs, outpatient visits, etc. were listed by the patients in the questionnaire.

Similarly, direct non-health costs related to transportation to health care providers and lodging expenses, stemming both from the patient and the caregiver, were also collected.

Costs for formal and informal assistance were estimated using the proxy-good method which values the care provided by the informal caregiver considering that if he/she did not provide these services, their presence would have to be substituted by another person who could provide them and thus considering hourly wage of formal caregiver as reported from Eurostat [13] and hours of assistance needed as listed by patients in the questionnaire.

Indirect costs were made up of productivity losses due to job loss or absenteeism and estimated using the human capital approach, considering working days lost declared by patients and hourly wage as from annual net earnings for Italy from Eurostat [13].

Costs over a 1-year period were estimated as twice the cost estimated for the 6 months from data collected from the questionnaire. All costs were referred to Euro 2022.

### Statistical analysis

Missing data about resources used data were replaced by the respective group median. The median instead of the arithmetic mean was used to avoid an overestimation of costs due to a small number of extremely high values.

Direct, indirect, and total costs are presented in aggregate and also the individual components of cost items as well as the associated use of resources are highlighted. Due to the skewed distribution of both resource use and costs, both the mean [minimum; maximum] and the median [25th–75th percentile] value were used to summarize data, while comparison between and among groups was performed using non-parametric tests, respectively, the Mann–Whitney test and Kruskal–Wallis test.

To assess the effect of time (years) since diagnosis and age of disease onset on overall costs, also adjusting for age, generalized linear model (GLM), or a two-part model were used. In details, as in the overall sample and among employed a large proportion of patients reported estimated costs equal to zero, the two-part model was used as it allows to properly model data using a first part modelling the probability of resulting in costs equal to zero, and a second part given by a GLM for cost conditional on resulting in costs different from zero. In the present study, a probit model was used for the first part and a generalized linear model with gamma distribution and log link for the second part. In all models robust standard errors were used to account for the nature of data with patients clustered into geographical areas. *P* < 0.05 was considered statistically significant. All analyses were conducted using STATA 17.

## Results

A total of 207 patients were considered in the present study, patients were mainly female (*n* = 139, 67.2%) and more than half (*n* = 137, 66.2%) aged between 31 and 50 years. A detailed description of patients’ characteristics is reported in Table S2.

Of note, among the patients included in the study, 131 (63.3%) of them declared to be employed, and among them, more than half (*n* = 72, 55%) stated that they need to change type and/or working hours because of BS.

Table 1 shows resource use related to both healthcare and non-healthcare resources detailing the number of patients declaring the use of the specific resource collected and the mean and median use per patient over a year. Of note, among healthcare resources, almost all patients declared to have specialists’ visits and imaging/lab exams (89.3% and 90.3%, respectively) in a year, with an average of about 1 visit or test per months. Similarly, the majority of patients were prescribed with drugs for BS (87.0%) with a mean of more than three drugs assumed. On the other hand, a minority of patients had hospitalization or ED visits (31.9%).

**Table 1:** Resource use per patient/year

	Number of patients (%)	Utilization, events per year	
		Mean (min;max)	Median [25–75th percentile]
Hospital visits	36 (17.3%)	0.57 (0;24)	0 [0–0]
One-day hospital visits	1 (0.5%)	0.06 (0;13)	0 [0–0]
Hospital visits (1 day or ordinary)	38 (18.4%)	0.63 (0;24)	0 [0–0]
ED visits	66 (31.9%)	1.35 (0;14)	0[0–2]
Outpatient visits^*^	186 (89.9%)	11.83 (0;80)	10 [4–16]
Blood tests	186 (89.9%)	8.15 (0;60)	6 [4–10]
Magnetic resonance	97 (46.9%)	1.54 (0;20)	0 [0–2]
Chest X-ray	69 (33.3%)	0.96 (0;16)	0 [0–2]
Colonoscopy/esophagogastroduenoscopy	43 (20.8%)	0.45 (0;4)	0 [0–0]
Visual field exam	113 (54.6%)	1.78 (0;12)	2 [0–2]
Ultrasounds	112 (54.1%)	1.91 (0;12)	2 [0–4]
Imaging/laboratory exams	187 (90.3%)	14.80 (0;74)	12 [6–20]
Drugs	180 (87.0%)	3.45 (0;7)	2 [1–5]
Out-of-pocket	119 (57.5%)	–	–
Transport/food/accommodation	107 (51.7)	–	–

	Number of patients (%)	Utilization, hours per year	
		Mean (min;max)	Median [25–75th percentile]
Formal assistance	137 (66.2%)	41.39 (0;30)	0 [0–0]
Informal assistance	127 (61.4%)	1007.19 (0;9,120)	288 [0–1,344]

	Number of patients (%)	Utilization, days per year	
		Mean (min;max)	Median [25–75th percentile]
Productivity loss	96 (46.4%)	20 (0;240)	0 [0–24]

*Including rheumatologic, oculist, dermatologic, gastroenterological, neurologic, gynaecologic, or other specialist visits

More than one half of patients declared out-of-pocket expenses (57.5%) and nearly half of patients incurred in expenses for food/accommodation/transportation because of BS-related visits or hospitalizations. Formal or informal assistance was required by, respectively, 66.2% and 61.4% of patients, while 46.4% of the sample lost working days because of BS with a mean of about 10 days lost/year.

**Fig. 1:**
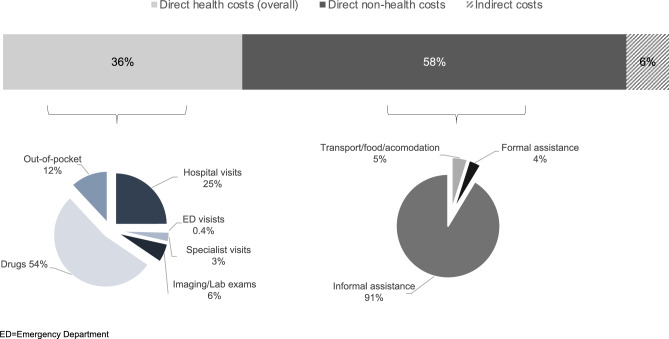
Contribution of the different cost’ components to the total cost

From the perspective of the Society, mean overall costs for BS patient were estimated to be 21,624 € (0;193,617) per patient/year. Direct non-health expenses were the main costs component accounting for 58% of the overall costs, followed direct health costs, 36%, while indirect costs because of productivity losses represented 6% of the overall costs, Fig. 1.

In details, mean direct non-health costs were estimated to be 12,689 € (0;108,209) per patient/year, almost doubling direct health costs that amounted to 7,892 € (0;85,409) per patient/year.

Among direct health costs, those related to prescribed drugs were the main driver representing 91% of the category and being estimated to be 4,228 € (0;31,627) per patient/year. Specialist visits, laboratory/imaging exams, and out-of-pocket expenses contributed, respectively, for the 3%, 6%, and 12%, while hospital visits represented one-quarter of direct health costs, despite only a minority of patients incurred costs related to hospital and ED admissions (Table 2 and Fig. 1).

**Table 2:** Mean and median costs (in Euro) per patient/year according to the different cost’ components

	Mean (min;max)	Median [25–75th percentile]
Hospital visits	1,971 (0;79,760)	0 [0–0]
ED visits	34 (0;350)	0 [0-50]
Specialist visits	244 (0;1,653)	207 [83-331]
Imaging/laboratory exams	473 (0;4,411)	363 [90-672]
Drugs	4228 (0;31,627)	252 [0-8,923]
Out-of-pocket	942 (0;12,092)	400 [0-1,200]
*Direct health costs*	*7892 (0;85,409)*	*4819 [925-12,304]*
Transport/food/accommodation	620 (0;14,800)	80 [0-540]
Formal assistance	476 (0;8,287)	0 [0-0]
Informal assistance	11,593 (0;104,971)	3315 [0-15,469]
*Direct non-health costs*	*12,689 (0;108,209)*	*3867 [0-15,884]*
Productivity loss	1381 (0;16,574)	0 [0-1,657]
*Direct non-health and indirect costs*	*14,071 (0;108,209)*	*5449 [691-18,232]*
*Overall*	*21,962 (0;193,617)*	*13,954 [3,767-30,824]*

Categories reported in italics represent the sum of the cost item above, similarly related numbers

ED = emergency department; Max = Maximum value; Min = minimum value

Mean costs for formal and informal assistance were 476 € (0;8,287) and 11,593 € (0;104,971) per patient/year, respectively, and mean costs for transport/food or accommodation were 620 € (0;14,800) per patient/year. Finally, indirect costs because of productivity loses were 1381 € (0;16,574) per patient/year (Table 2).

Being employed resulted in significantly lower overall costs (*p* = 0.006). Indeed, while not incurring in costs for productivity losses, patients declaring themselves to be not employed encountered significantly higher costs for informal assistance (*p* < 0.001), higher costs for transportation, food or accommodation (*p* < 0.001), and also higher costs for ED visits (*p* = 0.001), specialists visits (*p* = 0.002), imaging/laboratory exams (*p* = 0.008), and drugs (*p* = 0.003); see Table 3 and Table S3.

**Table 3:** Mean^*^ costs (in Euro) per patient/year according to working condition

	Non-employed (*N* = 76)	Employed (*N* = 131)	*P*
Hospital visits	2919 (0;79,760)	1421 (0;26,587)	0.181
ED visits	51 (0;350)	24 (0;350)	**0.001**
Specialist visits	296 (0;1,653)	214 (0;868)	**0.002**
Imaging/laboratory exams	555 (0;2,131)	426 (0;4,411)	**0.008**
Drugs	4242 (0;31,627)	4220 (0;18,907)	**0.003**
Out-of-pocket	1046 (0;6,600)	881 (0;12,092)	0.634
*Direct health costs*	*9108 (0;85,409)*	*7186 (0;29,897)*	0.108
Transport/food/accommodation	1065 (0;14,800)	362 (0;11,200)	** < 0.001**
Formal assistance	367 (0;8,287)	540 (0;8,287)	0.126
Informal assistance	20,166 (0;104,971)	6619 (0;83,424)	** < 0.001**
*Direct non-health costs*	*21,598 (0;108,209)*	*7521 (0;85,224)*	** < 0.001**
Productivity loss	–	2183 (0;16,574)	** < 0.001**
*Direct non-health and indirect costs*	*21,598 (0;108,209)*	*9704 (0;89,368)*	**0.041**
*Overall*	*30,706 (0;193,617)*	*16,890 (0;99,391)*	**0.006**

Categories reported in italics represent the sum of the cost item above, similarly related numbers

(*P* < 0.05) statistically significant values are highlighted in bold

^*^ Mean (minimum;maximum) costs are shown

ED = emergency department

Overall costs for BS did not differ significantly in relation to time since diagnosis (*p* = 0.887), despite direct health costs for specialist visits and imaging/laboratory exams were significantly higher among those diagnosed less than 1 year before the assessment (*p* < 0.001 and *p* = 0.008 respectively); see Table 4 and Table S4.

**Table 4:** Mean^*^ costs (in Euro) per patient/year according to years from diagnosis

	Overall sample (*N* = 207)			
Years since diagnosis	< 1 year (*N* = 29)	1–5 years (*N* = 62)	> 5 years (*N* = 116)	*P*
Hospital visits	4,813 (0;79,760)	858 (0;13,293)	1,855 (0;26,587)	0.079
ED visits	42 (0;250)	40 (0;350)	28 (0;350)	0.200
Specialist visits	369 (83;868)	268 (0;1,653)	200 (0;785)	** < 0.001**
Imaging/Lab exams	687 (0;2,131)	443 (0;1,966)	436 (0;4,411)	**0.006**
Drugs	1917 (0;31,627)	4355 (0;15,325)	4738 (0;31,602)	0.062
Out-of-pocket	956 (0;2,600)	1,387 (0;12,092)	700 (0;7,432)	0.065
*Direct health costs*	*8784 (331;85,409)*	*7,350 (0;25,214)*	*7958 (0;33,014)*	0.938
Transport/food/accommodation	666 (0;6,000)	806 (0;14,800)	510 (0;11,200)	0.825
Formal assistance	810 (0;8,287)	401 (0;7,735)	433 (0;8,287)	0.145
Informal assistance	17,422 (0;104,971)	11,861 (0;83,424)	9992 (0;86,739)	0.506
*Direct non-health costs*	*18,898 (0;108,209)*	*13,067 (0;85,224)*	*10,935 (0;86,739)*	0.661
Productivity loss	1343 (0;8,287)	1,185 (0;16,574)	1496 (0;16,574)	0.634
*Direct non-health and indirect costs*	*20,241 (0;108,209)*	*14,253 (0;89,368)*	*12,431 (0;86,739)*	0.530
*Overall*	*29,026 (331;193,617)*	*21,602 (0;99,391)*	*20,389 (0;93,145)*	0.909

	Non-employed (*N* = 76)			
Years since diagnosis	< 1 year (*N* = 12)	1–5 years (*N* = 23)	> 5 years (*N* = 41)	*P*
Hospital visits	8,308 (0;79,760)	1,445 (0;13,293)	2168 (0;15,768)	0.500
ED visits	67 (0;250)	63 (0;350)	40 (0;275)	0.256
Specialist visits	413 (124;868)	377 (0;1,653)	216 (0;6,61)	**0.010**
Imaging/Lab exams	828 (199;2,131)	576 (0;1,966)	462 (0;1,388)	0.068
Drugs	4,122 (0;31,627)	3,072 (0;11,615)	4,934 (0;31,602)	0.630
Out-of-pocket	1,139 (0;2,600)	1,436 (0;6,600)	800 (0;4,288)	0.374
*Direct health costs*	*14,877 (417;85,409)*	*6,969 (0;17,400)*	*8620 (23;33,014)*	0.891
Transport/food/accommodation	1,135 (0;6,000)	1,821 (0;14,800)	620 (0;4,000)	0.927
Formal assistance	852 (0;8,287)	384 (0;7,735)	216 (0;3,315)	0.409
Informal assistance	32,504 (0;104,971)	18,820 (0;62,983)	17,309 (0;86,739)	0.718
*Direct non-health costs*	*34,491 (0;108,209)*	*21,026 (0;77,783)*	*18,145 (0;86,739)*	0.796
Productivity loss	-	-	-	-
*Direct non-health and indirect costs*	*34,491 (0;108,209)*	*21,026 (0;77,783)*	*18,145 (0;86,739)*	0.796
*Overall*	*49,368 (537;193,617)*	*27,995 (0;94,480)*	*26,765 (23;93,145)*	0.864

	Employed (*N* = 131)			
Years since diagnosis	< 1 year (*N* = 17)	1–5 years (*N* = 39)	> 5 years (*N* = 75)	*P*
Hospital visits	2,346 (0;13,293)	511 (0;6,647)	1,684 (0;26,587)	0.112
ED visits	25 (0;175)	27 (0;300)	22 (0;350)	0.757
Specialist visits	338 (83;868)	204 (0;785)	192 (0;785)	**0.008**
Imaging/Lab exams	587 (0;1,319)	364 (0;1,728)	422 (0;4,411)	0.081
Drugs	360 (0;2,711)	5111 (0;15,325)	4631 (0;18,907)	0.063
Out-of-pocket	827 (0;2,400)	1357 (0;12,092)	646 (0;7,432)	0.200
*Direct health costs*	*4484 (331;15,917)*	*7574 (0;25,214)*	*7596 (0;29,897)*	0.688
Transport/food/accommodation	335 (0;2,600)	207 (0;1,800)	449 (0;11,200)	0.521
Formal assistance	780 (0;3,315)	411 (0;6,077)	552 (0;8,287)	0.305
Informal assistance	6776 (0;27,624)	7756 (0;83,424)	5993 (0;42,541)	0.653
*Direct non-health costs*	*7891 (0;29,281)*	*8374 (0;85,224)*	*6994 (0;46,541)*	0.593
Productivity loss	2291 (0;8,287)	1884 (0;16,574)	2313 (0;16,574)	0.214
*Direct non-health and indirect costs*	*10,182 (0;30,663)*	*10,258 (0;89,368)*	*9,307 (0;57,458)*	0.408
*Overall*	*14,666 (331;39,113)*	*17,833 (0;99,391)*	*16,904 (0;87,355)*	0.932

Categories reported in italics represent the sum of the cost item above, similarly related numbers

(*P* < 0.05) statistically significant values are highlighted in bold

*Mean (minimum;maximum) costs are shown

ED = emergency department

When distinguishing on the basis of working condition, costs for specialists visits still remained higher among those diagnosed less than 1 year before the assessment (*p* = 0.010 and *p* = 0.018 among non-employed and employed, respectively). On the other hand, no differences in costs for imaging/laboratory exams were found in the two groups according to time since diagnosis (*p* = 0.068 and *p* = 0.081 among non-employed and employed, respectively) being higher among non-employed, Table 4 and Table S4.

Overall costs were also similar in patients with different age at disease onset (*p* = 0.090). Nevertheless, patients with early disease onset (< 20 years) showed significant higher costs for specialist visits (*p* = 0.048) and for the overall direct non-health costs (*p* = 0.046), Table 5 and Table S5. Those differences were no more significant when distinguishing patients on the basis of working condition (*p* = 0.250 and *p* = 0.792 for specialist visits among non-employed and employed, respectively; *p* = 0.255 and *p* = 0.389) being costs for specialised visits for those experiencing first symptoms early significantly higher among non-employed (*p* = 0.040), Table 5 and Table S5.

**Table 5:** Mean^*^ costs (in Euro) per patient/year according to age at first symptoms

Overall sample (*N* = 207)				
Age at first symptoms	< 20 years (*N* = 92)	21–30 years (*N* = 57)	> 30 years (*N* = 58)	*P*
Hospital visits	2818 (0;79,760)	1326 (0;26,587)	1261 (0;13,293)	0.312
ED visits	38 (0;350)	38 (0;350)	24 (0;300)	0.215
Specialist visits	280 (0;1,653)	232 (0;868)	200 (0;785)	**0.048**
Imaging/Lab exams	514 (0;4,411)	421 (0;1,590)	460 (0;1,728)	0.713
Drugs	5204 (0;31,627)	2843 (0;18,401)	4041 (0;18,670)	0.396
Out-of-pocket	1016 (0;9,200)	1046 (0;12,092)	721 (0;7,432)	0.409
*Direct health costs*	*9869 (0;85,409)*	*5906 (0;29,897)*	*6,707 (0;21,772)*	0.098
Transport/food/accommodation	548 (0;6,000)	561 (0;11,200)	793 (0;14,800)	0.574
Formal assistance	589 (0;8,287)	431 (0;7,735)	343 (0;3,315)	0.457
Informal assistance	14,513 (0;104,971)	9,387 (0;50,276)	9,128 (0;88,949)	0.061
*Direct non-health costs*	*15,650 (0;108,209)*	*10,379 (0;51,608)*	*10,264 (0;92,054)*	**0.046**
Productivity loss	1691 (0;16,574)	1112 (0;16,574)	1154 (0;8,287)	0.729
*Direct non-health and indirect costs*	*17,341 (0;108,209)*	*11,492 (0;57,458)*	*11,418 (0;92,054)*	0.107
*Overall costs*	*27,210 (0;193,617)*	*17,397 (0;87,355)*	*18,124 (0;99,391)*	0.090

Non-employed (*N* = 76)				
Age at first symptoms	< 20 years (*N* = 36)	21–30 years (*N* = 21)	> 30 years (*N* = 19)	*P*
Hospital visits	4616 (0;79,760)	1067 (0;15,768)	1749 (0;13,293)	0.203
ED visits	56 (0;350)	43 (0;150)	50 (0;300)	0.944
Specialist visits	346 (0;1,653)	244 (0;496)	259 (0;785)	0.250
Imaging/Lab exams	587 (0;2,131)	500 (0;1,590)	554 (23;1,550)	0.784
Drugs	5065 (0;31,627)	3292 (0;18,401)	3734 (0;17,386)	0.986
Out-of-pocket	1029 (0;4,400)	1218 (0;6,600)	888 (0;3,694)	0.430
*Direct health costs*	*11,698 (417;85,409)*	*6364 (0;21,180)*	*7235 (23;21,772)*	0.476
Transport/food/accommodation	964 (0;6,000)	521 (0;5,200)	1,856 (0;14,800)	0.634
Formal assistance	276 (0;8,287)	487 (0;7735)	407 (0;3,315)	0.126
Informal assistance	25,330 (0;104,971)	13,641 (0;50,276)	17,592 (0;88,949)	0.416
*Direct non-health costs*	*26,570 (0;108,209)*	*14,649 (0;51,608)*	*19,855 (0;92,054)*	0.389
Productivity loss	–	–	–	–
*Direct non-health and indirect costs*	*26,570 (0;108,209)*	*14,649 (0;51,608)*	*19,855 (0;92,054)*	0.389
*Overall costs*	*38,268 (520;193,617)*	*21,013 (0;65,563)*	*27,090 (23;95,935)*	0.434

Employed (*N* = 131)				
Age at first symptoms	< 20 years (*N* = 56)	21–30 years (*N* = 36)	> 30 years (*N* = 39)	*P*
Hospital visits	1662 (0;19,940)	1477 (0;26,587)	1023 (0;6,647)	0.976
ED visits	25 (0;300)	35 (0;350)	12 (0;150)	0.339
Specialist visits	237 (0;785)	225 (0;868)	172 (0;537)	0.792
Imaging/Lab exams	467 (0;4,411)	375 (0;1,495)	414 (0;1,728)	0.304
Drugs	5294 (0;18,907)	2581 (0;16,851)	4190 (0;18,670)	0.600
Out-of-pocket	1008 (0;9,200)	945 (0;12,092)	640 (0;7,432)	0.204
*Direct health costs*	*8694 (0;29,589)*	*5639 (0;29,897)*	*6449 (0;19,744)*	0.118
Transport/food/accommodation	281 (0;4,000)	584 (0;11,200)	275 (0;3,400)	0.803
Formal assistance	789 (0;8,287)	399 (0;6,077)	312 (0;3,315)	0.641
Informal assistance	7560 (0;46,408)	6906 (0;40,884)	5004 (0;83,424)	0.887
*Direct non-health costs*	*8630 (0;48,618)*	*7889 (0;40,884)*	*5591 (0;85,224)*	0.255
Productivity loss	2778 (0;16,574)	1761 (0;16,574)	1716 (0;8,287)	0.182
*Direct non-health and indirect costs*	*11,408 (0;56,905)*	*9650 (0;57,458)*	*7307 (0;89,368)*	0.175
*Overall costs*	*20,102 (0;78,404)*	*15,288 (0;87,355)*	*13,756 (0;99,391)*	0.157

Categories reported in italics represent the sum of the cost item above, similarly related numbers

(*P* < 0.05) statistically significant values are highlighted in bold

*Mean (minimum;maximum) costs are shown

ED = emergency department

Results from the multivariate regression analyses suggested that the probability of incurring in overall costs equal to zero decreased as time from BS diagnosis is 1 year or more as compared to newly diagnosed patients (*p* < 0.001, for both those diagnosed 1–5 years before the interview and those being diagnosed more than 5 years before).

Conversely, among those incurring in expenses, costs decreased for those experiencing first symptoms between 21 and 30 years (*p* = 0.027) or later (*p* = 0.032) as compared to those having symptoms earlier, Table 6.

**Table 6:** Total costs: results of the multivariate regression analyses

	Overall sample (*N* = 207)		
	Coef. (Std.Err)	*P*	(95%CI)
*Probit (probability of not generating costs)*			
Age < = 40 years	*(ref)*		
Age > 40 years	0.130 (0.291)	0.654	(− 0.439;0.700)
Years since diagnosis < 1	*(ref)*		
Years since diagnosis 1–5	− 4.184 (0.388)	** < 0.001**	(− 4.946;− 3.423)
Years since diagnosis > = 6	− 3.592 (0.377)	** < 0.001**	(− 4.331;− 2.853)
Years of first symptoms < = 20	*(ref)*		
Years of first symptoms 21–30	− 0.339 (0.435)	0.435	(− 1.191;0.513)
Years of first symptoms > 30	0.193 (0.231)	0.403	(− 0.260;0.646)
Intercept	5.597 (0.375)	** < 0.001**	(4.862;6.333)
*GLM*			
Age < = 40 years	*(ref)*		
Age > 40 years	0.034 (0.108)	0.750	(− 0.177;0.246)
Years since diagnosis < 1	*(ref)*		
Years since diagnosis 1–5	− 0.163 (0.190)	0.391	(− 0.536;0.210)
Years since diagnosis > = 6	− 0.368 (0.366)	0.315	(− 1.086;0.350)
Years of first symptoms < = 20	*(ref)*		
Years of first symptoms 21–30	− 0.424 (0.191)	**0.027**	(− 0.798;− 0.049)
Years of first symptoms > 30	− 0.472 (0.219)	**0.032**	(− 0.902;− 0.042)
Intercept	10.447 (0.335)	** < 0.001**	(9.791–11.104)

	Non-employed (*N* = 76)^*^		
	Coef. (Std.Err)	*P*	(95%CI)
*GLM*			
Age < = 40 years	*(ref)*		
Age > 40 years	− 0.161 (0.138)	0.241	(− 0.431;0.108)
Years since diagnosis < 1	*(ref)*		
Years since diagnosis 1–5	− 0.279 (0.419)	0.506	(− 1.101;0.543)
Years since diagnosis > = 6	− 0.543 (0.624)	0.384	(− 1.766;0.680)
Years of first symptoms < = 20	*(ref)*		
Years of first symptoms 21–30	− 0.530 (0.343)	0.123	(− 1.203;0.143)
Years of first symptoms > 30	− 0.295 (0.336)	0.379	(− 0.953;0.363)
Intercept	11.125 (0.325)	** < 0.001**	(10.488–11.761)

	Employed (*N* = 131)		
	Coef. (Std.Err)	*P*	(95%CI)
*Probit (probability of not generating costs)*			
Age < = 40 years	*(ref)*		
Age > 40 years	0.08 (0.241)	0.740	(− 0.392;0.552)
Years since diagnosis < 1	*(ref)*		
Years since diagnosis 1–5	− 4.358 (0.340)	** < 0.001**	(− 5.024;− 3.693)
Years since diagnosis > = 6	− 3.779 (0.318)	** < 0.001**	(− 4.402;− 3.157)
Years of first symptoms < = 20	*(ref)*		
Years of first symptoms 21–30	− 0.291 (0.341)	0.394	(− 0.959;0.377)
Years of first symptoms > 30	0.226 (0.308)	0.463	(− 0.377;0.828)
Intercept	5.665 (0.385)	** < 0.001**	(4.911;6.419)
*GLM*			
Age < = 40 years	*(ref)*		
Age > 40 years	0.232 (0.182)	0.202	(− 0.125;0.590)
Years since diagnosis < 1	*(ref)*		
Years since diagnosis 1–5	0.156 (0.222)	0.483	(− 0.280;0.592)
Years since diagnosis > = 6	0.021 (0.154)	0.892	(− 0.280;0.322)
Years of first symptoms < = 20	*(ref)*		
Years of first symptoms 21–30	− 0.195 (0.059)	**0.001**	(− 0.311;− 0.079)
Years of first symptoms > 30	− 0.415 (0.290)	0.153	(− 0.983;0.154)
Intercept	9.519 (0.247)	** < 0.001**	(9.035–10.003)

(*P* < 0.05) statistically significant values are highlighted in bold

*Only the GLM model is shown as just 1 patient resulted in overall costs equal to zero

Similar findings emerged among the subgroups of patients declaring themselves as workers, while no impact of years since diagnosis or age of first symptoms was found among non-workers, Table 6.

When focusing on direct health costs in the overall sample, time since diagnosis and age of first symptoms, respectively, had a significant impact on the probability of incurring in costs and on the magnitude of costs. Among those declaring themselves to be non-workers direct health costs decreased for higher age, i.e., >  = 40 years old (*p* = 0.040); while among workers results highlighted a significant and negative effect of time since diagnosis higher than 1 year on the probability of not incurring in costs (*p* < 0.001 both for those diagnosed 1 to 5 years and more than 5 years before the assessment), see Table S6.

To what concern direct non-health costs, in the overall sample being diagnosed 1 to 5 years before the assessment and having first symptoms after the age of 30 significantly decreased the probability of not incurring in costs (*p* = 0.024 and *p* < 0.001, respectively). Among non-employed, having first symptoms after the age of 30 decreased the probability of not encountering direct health costs (*p* < 0.001), while being aged over 40 increased that probability (*p* < 0.001). None of the variables explored had a significant impact on the magnitude of direct non-health costs in the two subgroups, see Table S6.

Finally, among employed respondents, the probability of incurring in costs for productivity losses decreased among those diagnose 6 years before the assessment or more as compared with those with recent diagnosis (*p* = 0.001), while for those incurring in costs, the amount of indirect costs increased for those aged over 40 (as compared to younger patients, *p* < 0.001) and decreased those diagnosed 6 year before the assessment or more (*p* = 0.040).

## Discussion

Despite COI studies might support management and monitoring of costs, while also help evaluation of the impact of both policies and health technologies, at present, literature of COI studies among RDs and in particular among BS patients is scarce [14, 15].

The general aim of the present study was the evaluation of the cost of illness in BS, and to the best of our knowledge, this is the first study in Europe, thus representing a tangible contribution towards the understanding of the impact of a complex and rare disease such as BS. Specific aims of the study were also the assessment of the contribution of the different cost components on the overall economic burden and the evaluation of costs according to disease history, all evaluated taking into account possible diverse effects between the subgroups of employees and not-employees.

The relapsing nature of BS can cause remission and exacerbations of symptoms over time, and although different treatments are available, BS can still represent an important cause of morbidity due to the different subsets of organ involvement [4]. Accordingly, BS may have a significant impact on the lives of patients and their families, worsening both the psychological status, their quality of life, and having a considerable impact on the ability to perform daily life activities [16–19].

Indeed, results of the present study highlighted that direct non-health costs, almost represented by costs for informal assistance, underpin the social economic burden of BS, accounting for more than half of total costs; followed direct costs and finally costs for productivity loss. Moreover, the relative weight of informal assistance is even higher among non-employees. This subgroup, that was found to incur in higher costs as compared to the employees counterpart, also showed significantly higher costs for outpatient visits, imaging and laboratory exams, drugs, ED visits, and thus probably representing a subgroup of patients with more severe disease.

Despite RD have progressively been acknowledged as an important public health issue and policies have been set accordingly, particularly trying to improve availability and access to orphan drugs [20–23] and specialised healthcare [24], at present, many RD have no or no effective treatment available or, even if treatments exist, there is no guarantee of improvement in life expectancy or quality of life, with consequent impact on patients and their families as well as societal costs [25].

As the findings of the present study showed and as increasing recent evidence highlighted, it is important to go beyond the payer standpoint when evaluating, in Health Technology Assessment (HTA) perspective, new treatments devoted to RD as understanding hidden social costs are of paramount importance to assess the overall value of interventions [15, 21, 26].

Moreover, the burden of informal care found also in the present study emphasises the need to increase resources dedicated to social support for families, and to improve visibility and social protection of informal caregivers, while also improving the social recognition of their work [25].

Among direct health costs, costs for prescribed drugs prevailed, accounting for more than half of that cost component. Indeed, most of the patients involved in the present study declared to use more than one drug to manage BS and related comorbidities and, in some cases, costly biological drugs are used, thus partially explaining the contribution of those costs.

Despite differences limiting the possibility of comparison (i.e., different place of recruitment, specificity of the INHS, methods adopted to ascertain resource use, etc.), in a previous COI study conducted among BS treated in a tertiary centre in Turkey [7], drug costs were found to provide the main contribution to the amount of direct health costs.

The relative weight of the other components of direct health costs is narrow and needs to be interpreted also taking into account the large variability found in costs data. Indeed, even the amount of average costs related to hospital admission is largely driven by extremely high costs incurred by a minority of patients, whereas no costs for hospital admissions were estimated for the large part of the sample.

On the other hand, most of the patients had outpatient visits and imaging or laboratory exams in a year, despite associated costs have a relatively low contribution to the overall direct health costs in view of the fact that the Italian National Health System (INHS) foresees, upon BS diagnosis, the attribution of a disease-specific code that allows the partial or total coverage (depending on the region of residence) of most healthcare-related expenses (e.g., specialised consultations, examinations, etc.) needed to the management of BS at the onset and during the follow-up.

Despite that, more than half of the patients incurred out-of-pocket expenses and these could be probably related to services not (or partially) covered or services for which supply is limited in the national or regional health system, i.e., physiotherapy, psychological, or psychiatric support. Again, the burden of out-of-pocket expenses possibly highlighted the need to enlarge the access to the types of services that may be needed for the management of BS and other RDs.

When trying to disentangle the impact of disease history, in terms of both years since diagnosis and age at first symptoms, on overall costs using multivariate regression models, findings showed a decreasing probability of not incurring in costs over a year in patients that received a BS diagnosis from 1 year or more, compared to newly diagnosed patients probably in view of the fact that in recently diagnosed patients, more clinical evaluations are necessary to complete the clinical picture, what is sometimes referred as the diagnostic odyssey. Moreover, previous studies also highlighted that the severity of the diseases diminishes with the passage of time [5, 19]. On the other hand, among those experiencing costs, these decreased in patients experiencing first symptoms between 21 and 30 years or > 30 years compared to those having symptoms earlier. This can be probably explained both by the fact that even more clinical attention and more examinations might be prescribed due to the young age of patients manifesting such complex symptoms and because early disease onset generally runs a more severe course [19] with progressive accrual of disease manifestations coupled with an increased difficulty in accepting the disease. Those figures could also partially explain the lower probability of incurring in costs for productivity loss and even lower indirect costs among employees not recently diagnosed.

Similar findings were found when focusing on direct health costs and also when restricting the analysis on the subgroups of employees.

Some limitations of the present study need to be acknowledged. First of all, the study design foresees recruitment of participants from social media limiting the representativeness of the study sample because of a possible selection bias caused by the willingness to participate into the study and the impossibility to participate for BS patients that do not have access to social media; moreover, the retrospective collection of data that may be subject to recall bias.

Another important limitation is related to the unavailability of data about disease severity and/or organ involvement that, as in previous studies [7, 27], limit the possibility to evaluate costs accordingly.

Finally, due to the study design, it is not possible to quantify the excess of costs induced by BS compared to the general population, nor to compare the economic impact estimated for BS with that of similar diseases.

Despite limitations, the present study updates and expands the single COI study available on BS [7] by offering a comprehensive overview of the economic consequences imposed by BS in a societal perspective. Moreover, the results of our analysis provide insights into the distribution of the different costs components related to BS.

Future studies should devote efforts to improve epidemiological information BS, that is currently scarce and heterogeneous, while also enabling routinely collection of economic facts, to make suitable estimation of the economic impact of BS on national expenditures. Recent initiatives go in that direction [28].

### Supplementary Information

Below is the link to the electronic supplementary material.

Supplementary file1 An English translation of the questionnaire used in the study is reported as Online resource 1. Supplementary data show details of costs used to value healthcare resources (Table S1) and details of patients characteristics (Table S2). Median costs according to working condition (Table S3), years from diagnosis (Table S4), age at first symptoms (Table S5) as well as results of the multivariate regression model for directs health costs, direct non health and indirect costs according to working condition (Table S6) are also reported as supplementary material. (DOCX 48 KB)

## Data Availability

Data would be available upon request to authors.
