# Supplementary material for: A cost-of-illness study of Behçet syndrome in Italy
Source: Eur J Health Econ. 2023 May 22;25(3):411–22. doi: 10.1007/s10198-023-01593-8 (PMC10973046; doi:10.1007/s10198-023-01593-8)
Supplement: Supplementary file 1 — Supplementary file1 An English translation of the questionnaire used in the study is reported as Online resource 1. Supplementary data show details of costs used to value healthcare resources (Table S1) and details of patients characteristics (Table S2). Median costs according to working condition (Table S3), years from diagnosis (Table S4), age at first symptoms (Table S5) as well as results of the multivariate regression model for directs health costs, direct non health and indirect costs according to working condition (Table S6) are also reported as supplementary material. (DOCX 48 KB) [file 10198_2023_1593_MOESM1_ESM.docx]

**Supplementary material**

**Table S1. Details of unit cost considered to value direct health costs for specialist visits and imaging/laboratory exams**

| ICD code and service description | Unit cost (€) |
| --- | --- |
| 89.7-Other specialist visit | 20.66 |
| 89.26-Gynecologic visit | 20.66 |
| 94.12.1-Psychiatric visit | 12.91 |
| 89.13-Neurologic visit | 20.66 |
| 91.49.2-Venous blood sampling | 2.58 |
| 90.62.2-Haemochrome | 3.17 |
| 88.91.1-Magnetic resonance imaging of the brain | 166.58 |
| 87.44.1-Chest X-ray | 15.49 |
| 88.76.1-Full abdomen ultrasound | 60.43 |
| 88.72.1-Cardiac ultrasound | 51.65 |
| 88.79.2-Osteoarticular ultrasound | 32.54 |
| 45.23-Colonoscopy | 86.80 |
| 45.13-Esophagogastroduenoscopy | 56.81 |
| 95.05-Visual field exam | 16.78 |

**Table S2. Main characteristics of the study population**

|  |  | **Number of patients (%)** |
| --- | --- | --- |
| **Gender** | **Female** | 139  (67.15%) |
|  | **Male** | 68 (32.85%) |
| **Age** | **18-20 years** | 8 (3.86%) |
|  | **21-30 years** | 30 (14.49%) |
|  | **31-40 years** | 66 (31.88%) |
|  | **41-50 years** | 71 (34.30%) |
|  | **51-60 years** | 26 (12.56%) |
|  | **61-70 years** | 6 (2.90%) |
| **Age at first symptoms** | **0-10 years** | 37 (17.87%) |
|  | **11-20 years** | 55 (26.57%) |
|  | **21-30 years** | 57 (27.54%) |
|  | **31-40 years** | 44 (21.26%) |
|  | **41-50 years** | 13 (6.28%) |
|  | **51-60 years** | 1 (0.48%) |
| **Time since diagnosis** | **<1 years** | 29 (14.01%) |
|  | **1-5 years** | 62 (29.95%) |
|  | **6-10 years** | 41 (19.81%) |
|  | **11-15 years** | 28 (13.53%) |
|  | **16-20 years** | 21 (10.14%) |
|  | **>=21 years** | 26 (12.56%) |
| **Marital status** | **Single** | 61 (29.47%) |
|  | **Married** | 96 (46.38%) |
|  | **Cohabitant** | 27 (13.04%) |
|  | **Divorced** | 22 (10.63%) |
|  | **Widow** | 1 (0.48%) |
| **Education** | **None** | 1 (0.48%) |
|  | **High school diploma** | 109 (52.66%) |
|  | **Secondary school diploma** | 33 (15.94%) |
|  | **Degree** | 42 (20.29%) |
|  | **Postgraduate degree** | 22 (10.63%) |
| **Working condition** | **Housewife** | 11 (5.31%) |
|  | **Unemployed** | 24 (11.59%) |
|  | **Unable to work** | 17 (8.21%) |
|  | **Retired** | 9 (4.35%) |
|  | **Student** | 15 (7.25%) |
|  | **Employed** | 131 (63.29%) |
| **Full-time/Part-time worker** | **Part-time** | 37 (28.24%) |
|  | **Full time** | 94 (71.76%) |
| **Need to change working life** | **No** | 71 (34.30%) |
|  | **Yes** | 136 (65.70%) |

**Table S3. Median^*^ costs (in Euro) per patient/year according to working condition**

|  | Non-employed (N=76) | Employed (N=131) |
| --- | --- | --- |
| Hospital visits | 0 [0-0] | 0 [0-0] |
| ED visits | 0 [0-100] | 0 [0-0] |
| Specialist visits | 248 [124-413] | 165 [83-331] |
| Imaging/Laboratory exams | 496 [218-746] | 269 [54-564] |
| Drugs | 519 [15-6,755] | 189 [0-10,057] |
| Out-of-pocket | 503 [29-1,652] | 271 [0-1,178] |
| *Direct health costs* | ***4,489 [1,518-14,501]*** | ***5,052 [728-12,128]*** |
| Transport/food/accommodation | 370 [0-1,120] | 0 [0-400] |
| Formal assistance | 0 [0-0] | 0 [0-0] |
| Informal assistance | 7,458 [0-32,873] | 1,934 [0-8,840] |
| *Direct non-health costs* | 10,277 [270-35,020] | 2,822 [0-10,058] |
| Productivity loss | - | 1,381 [0-2,417] |
| *Direct non-health and indirect costs* | ***10,277 [270-35,020]*** | ***3,915 [814-14,019]*** |
| *Overall* | ***19,220 [5,989-44,666]*** | ***12,917 [3,197-22,077]*** |

* Median (25^th^-75^th^ percentile) costs are shown

ED=emergency department

**Table S4. Median^*^ costs (in Euro) per patient/year according to years from diagnosis**

|  | Overall sample (N=207) | | | |
| --- | --- | --- | --- | --- |
| Years since diagnosis | **<1 year (N=29)** | **1-5 years (N=62)** | | **>5 years (N=116)** |
| Hospital visits | 0 [0-6,647] | 0 [0-0] | | 0 [0-0] |
| ED visits | 0 [0-50] | 0 [0-50] | | 0 [0-50] |
| Specialist visits | 331 [207-455] | 186 [83-413] | | 165 [83-289] |
| Imaging/Lab exams | 699 [241-888] | 284 [45-672] | | 355 [103-563] |
| Drugs | 95 [0-576] | 599 [28-9,041] | | 388 [0-9,892] |
| Out-of-pocket | 900 [180-1,785] | 379 [0-1,557] | | 293 [0-935] |
| *Direct health costs* | ***3,574 [1327-9,628]*** | ***6306 [1070-12304]*** | | ***5,359 [899-13,117]*** |
| Transport/food/accommodation | 0 [0-700] | 30 [0-400] | | 140 [0-600] |
| Formal assistance | 0 [0-1,105] | 0 [0-0] | | 0 [0-0] |
| Informal assistance | 5,525 [0-16,574] | 3,453 [0-17,679] | | 2,762 [0-13,536] |
| *Direct non health costs* | ***5,525 [120-16,574]*** | ***3,853 [0-18,519]*** | | ***3,505 [0-14,741]*** |
| Productivity loss | 1,105 [0-2,072] | 0 [0-1,657] | | 0 [0-1,381] |
| *Direct non health and indirect costs* | ***9,340 [1,381-18,232]*** | ***4,205 [552-19,164]*** | | ***4,647 [663-16,574]*** |
| *Overall* | ***15,791 [6,237-24,019]*** | ***13,233 [3,453-30,481]*** | | ***14,704 [4,012-32,327]*** |
|  | **Non-employed (N=76)** | | | |
| Years since diagnosis | **<1 year (N=12)** | **1-5 years (N=23)** | **>5 years (N=41)** | |
| Hospital visits | 0 [0-3,323] | 0 [0-0] | | 0 [0-6,647] |
| ED visits | 25 [0-100] | 50 [0-50] | | 0 [0-50] |
| Specialist visits | 351 [248-537] | 372 [124-496] | | 207 [124-331] |
| Imaging/Lab exams | 708 [513-968] | 423 [91-869] | | 453 [208-649] |
| Drugs | 147 [6-789] | 1,330 [135-5,686] | | 393 [16-9,687] |
| Out-of-pocket | 992 [400-1,839] | 400 [0-1,900] | | 486 [0-960] |
| *Direct health costs* | ***4,345 [2,625-15,711]*** | ***5020 [1,815-11,721]*** | | ***4,413 [1,321-16,307]*** |
| Transport/food/accommodation | 310 [0-1,750] | 200 [0-4240] | | 380 [0-700] |
| Formal assistance | 0 [0-414] | 0 [0-0] | | 0 [0-0] |
| Informal assistance | 12,707 [0-71,546] | 5,525 [0-42,541] | | 8287 [0-27,624] |
| *Direct non health costs* | ***12,957 [60-73,349]*** | ***9,925 [140-46,608]*** | | ***10,630 [560-29,024]*** |
| Productivity loss | ***-*** | ***-*** | | ***-*** |
| *Direct non health and indirect costs* | ***12,957 [60-73,349]*** | ***9,925 [140-46,608]*** | | ***10,630 [560-29,024]*** |
| *Overall* | ***18,295 [7,933-82,779]*** | ***15,890 [7,740-48,438]*** | | ***19,612 [4,513-41,396]*** |
|  | **Employed (N=131)** | | | |
| Years since diagnosis | **<1 year (N=17)** | **1-5 years (N=39)** | **>5 years (N=75)** | |
| Hospital visits | 0 [0-6,647] | 0 [0-0] | | 0 [0-0] |
| ED visits | 0 [0-50] | 0 [0-0] | | 0 [0-0] |
| Specialist visits | 331 [165-413] | 165 [41-289] | | 124 [83-289] |
| Imaging/Lab exams | 625 [176-888] | 216 [23-520] | | 240 [54-531] |
| Drugs | 42 [0-216] | 360 [0-11,572] | | 252 [0-10,320] |
| Out-of-pocket | 593 [0-1,385] | 359 [0-1400] | | 240 [0-910] |
| *Direct health costs* | ***2,848 [1,247-8,305]*** | ***8,115 [654-12,325]*** | | ***7,083 [624-12,201]*** |
| Transport/food/accommodation | 0 [0-400] | 0 [0-400] | | 0 [0-360] |
| Formal assistance | 0 [0-1,657] | 0 [0-0] | | 0 [0-0] |
| Informal assistance | 4420 [0-9,945] | 552 [0-8,840] | | 1,934 [0-7,735] |
| *Direct non health costs* | ***4,420 [400-11,050]*** | ***1,052 [0-9,945]*** | | ***2,992 [0-9,477]*** |
| Productivity loss | 1,865 [1,381-2,762] | 1,243 [0-2,072] | | 1,381 [0-2,072] |
| *Direct non health and indirect costs* | ***7,942 [1,781-14,019]*** | ***2,822 [552-12,983]*** | | ***4,067 [691-14,161]*** |
| *Overall* | ***13,954 [5,390-19,661]*** | ***12,822 [2,550-28,000]*** | | ***12,847 [3,767-21,401]*** |

*Median [25^th^-75^th^ percentile] costs are shown

ED=emergency department

**Table S5. Median^*^ costs (in Euro) per patient/year according to age at first symptoms**

| Overall sample (N=207) | | | | |
| --- | --- | --- | --- | --- |
| Age at first symptoms | **<20 years (N=92)** | **21-30 years (N=57)** | **>30 years (N=58)** | |
| Hospital visits | 0 [0-0] | 0 [0-0] | | 0 [0-0] |
| ED visits | 0 [0-50] | 0 [0-50] | | 0 [0-0] |
| Specialist visits | 248 [124-331] | 165 [83-331] | | 145 [83-248] |
| Imaging/Lab exams | 375 [119-737] | 388 [57-625] | | 342 [69-697] |
| Drugs | 509 [0-10,715] | 197 [0-4,356] | | 234 [0-8,713] |
| Out-of-pocket | 412 [0-1,344] | 400 [0-1,393] | | 303 [0-953] |
| *Direct health costs* | ***8,910 [942-14,745]*** | ***3,216 [890-9,170]*** | | ***4,066 [1,126-11,721]*** |
| Transport/food/accommodation | 100 [0-520] | 180 [0-500] | | 0 [0-600] |
| Formal assistance | 0 [0-0] | 0 [0-0] | | 0 [0-0] |
| Informal assistance | 5525 [0-18,508] | 2,762 [0-15,469] | | 0 [0-8,287] |
| *Direct non health costs* | ***6,680 [240-20,389]*** | ***3,442 [60-16,951]*** | | ***952 [0-10,630]*** |
| Productivity loss | 0 [0-1,519] | 0 [0-1381] | | 345 [0-1,865] |
| *Direct non health and indirect costs* | ***8,868 [870-22,071]*** | ***4,067 [691-18,232]*** | | ***3,000 [414-11,342]*** |
| *Overall costs* | ***18,449 [4,548-39,217]*** | ***11,544 [4,256-23,264]*** | | ***12,439 [2,904-22,016]*** |
| Non-employed (N=76) | | | | |
| Age at first symptoms | **<20 years (N=36)** | **21-30 years (N=21)** | **>30 years (N=19)** | |
| Hospital visits | 0 [0-6,647] | 0 [0-0] | | 0 [0-0] |
| ED visits | 0 [0-100] | 0 [0-100] | | 0 [0-50] |
| Specialist visits | 289 [165-413] | 248 [165-331] | | 165 [83-413] |
| Imaging/Lab exams | 513 [274-777] | 464 [229-631] | | 453 [68-1,057] |
| Drugs | 388 [6-9,127] | 682 [135-5,686] | | 860 [0-4,329] |
| Out-of-pocket | 553 [164-1,839] | 579 [0-1,518] | | 400 [0-1,084] |
| *Direct health costs* | ***8,910 [1,355-16,631]*** | ***4,100 [1,647-9,170]*** | | ***3,880 [1,679-14,023]*** |
| Transport/food/accommodation | 450 [0-1,400] | 200 [0-400] | | 380 [0-2,840] |
| Formal assistance | 0 [0-0] | 0 [0-0] | | 0 [0-552] |
| Informal assistance | 11,050 [0-49,723] | 3,591 [0-18,784] | | 6,630 [0-27,624] |
| *Direct non health costs* | ***12,070 [1,320-52,313]*** | ***3,991 [400-19,456]*** | | ***10,630 [0-29,921]*** |
| Productivity loss | - | - | | - |
| *Direct non health and indirect costs* | ***12,070 [1,320-52,313]*** | ***3,991 [400-19,456]*** | | ***10,630 [0-29,921]*** |
| *Overall costs* | ***23,609 [5,410-61,332]*** | ***18,272 [7,740-34,744]*** | | ***17,514 [5,742-41,396]*** |
| Employed (N=131) | | | | |
| Age at first symptoms | **<20 years (N=56)** | **21-30 years (N=36)** | **>30 years (N=39)** | |
| Hospital visits | 0 [0-0] | 0 [0-0] | | 0 [0-0] |
| ED visits | 0 [0-0] | 0 [0-50] | | 0 [0-0] |
| Specialist visits | 207 [103-331] | 145 [83-331] | | 124 [83-248] |
| Imaging/Lab exams | 276 [50-567] | 187 [6-562] | | 274 [69-625] |
| Drugs | 604 [0-11,596] | 126 [0-2,644] | | 181 [0-9,041] |
| Out-of-pocket | 330 [0-1,250] | 315 [0-1121] | | 263 [0-917] |
| *Direct health costs* | ***8,761 [744-13,815]*** | ***3,161 [617-9,849]*** | | ***7,802 [663-11,667]*** |
| Transport/food/accommodation | 0 [0-250] | 140 [0-640] | | 0 [0-200] |
| Formal assistance | 0 [0-1,105] | 0 [0-0] | | 0 [0-0] |
| Informal assistance | 3,039 [0-12,569] | 2,486 [0-8,840] | | 0 [0-4,420] |
| *Direct non health costs* | ***4,144 [100-14,426]*** | ***3,201 [0-11,125]*** | | ***500 [0-4,420]*** |
| Productivity loss | 1,312 [0-3,798] | 1,243 [0-2,072] | | 1,381 [276-2,072] |
| *Direct non health and indirect costs* | ***6,971 [732-16,574]*** | ***4,105 [829-13,197]*** | | ***2,181 [914-8,563]*** |
| *Overall costs* | ***17,042 [3,642-30,991]*** | ***11,363 [3,726-18,807]*** | | ***11,667 [2,709-17,960]*** |

*Median (25^th^-75^th^ percentile) costs are shown

ED=emergency department

**Table S6. Results of the multivariate regression model for directs health costs, direct non health and indirect costs according to working condition**

| Overall sample (N=207) | | | | | | | |
| --- | --- | --- | --- | --- | --- | --- | --- |
|  | **Direct health costs** | | | **Direct non health costs** | | | |
|  | **Coef. (Std.Err)** | **P-value** | **(95%CI)** | | **Coef. (Std.Err)** | **P-value** | **(95%CI)** |
| *Probit (Prob. of not incurring in costs)* | | | | | | | |
| Age <=40 years | *(ref.)* | | | | | | |
| Age >40 years | 0.442 (0.428) | 0.302 | (-0.396;1.280) | | 0.408 (0.258) | 0.113 | (-0.097;0.913) |
| Time since diagnosis <1 years | *(ref.)* | | | | | | |
| Time since diagnosis 1-5 years | **-4.310 (0.362)** | **<0.001** | **(-5.020;-3.600)** | | **-0.134 (0.059)** | **0.024** | **(-0.25;-0.018)** |
| Time since diagnosis >=6 years | **-3.875 (0.212)** | **<0.001** | **(-4.291;-3.458)** | | -0.218 (0.287) | 0.449 | (-0.781;0.346) |
| Age of first symptoms <=20 years | *(ref.)* | | | | | | |
| Age of first symptoms 21-30 years | 0.076 (0.304) | 0.803 | (-0.520;0.672) | | -0.138 (0.231) | 0.550 | (-0.591;0.315) |
| Age of first symptoms >30 years | 0.465 (0.330) | 0.159 | (-0.182;1.113) | | **-0.678 (0.154)** | **<0.001** | **(-0.981;-0.376)** |
| Intercept | **4.973 (0.500)** | **<0.001** | **(3.993;5.952)** | | 0.433 (0.475) | 0.362 | (-0.498;1.364) |
| *Generalized Linear Model* | | | | | | | |
| Age <=40 years | *(ref.)* | | | | | | |
| Age >40 years | -0.140 (0.128) | 0.275 | (-0.391;0.111) | | -0.147 (0.102) | 0.147 | (-0.346;0.052) |
| Time since diagnosis <1 years | *(ref.)* | | | | | | |
| Time since diagnosis 1-5 years | 0.026 (0.263) | 0.920 | (-0.489;0.542) | | -0.200 (0.276) | 0.469 | (-0.741;0.341) |
| Time since diagnosis >=6 years | 0.023 (0.351) | 0.949 | (-0.666;0.711) | | -0.513 (0.342) | 0.133 | (-1.183;0.156) |
| Age of first symptoms <=20 years | *(ref.)* | | | | | | |
| Age of first symptoms 21-30 years | **-0.529 (0.178)** | **0.003** | **(-0.877;-0.180)** | | -0.377 (0.196) | 0.054 | (-0.761;0.007) |
| Age of first symptoms >30 years | **-0.397 (0.117)** | **0.001** | **(-0.626;-0.167)** | | -0.218 (0.300) | 0.466 | (-0.805;0.369) |
| Intercept | **8.744 (0.436)** | **<0.001** | **(-0.545-0.513)** | | **10.452 (0.327)** | **<0.001** | (9.812;11.093) |
| Non-employed (N=76) | | | | | | | |
|  | **Direct health costs** | | | **Direct non health costs** | | | |
|  | **Coef. (Std.Err)** | **P-value** | **(95%CI)** | | **Coef. (Std.Err)** | **P-value** | **(95%CI)** |
| *Probit (Prob. of not incurring in costs)^*^* | | | | | | | |
| Age <=40 years | *(ref.)* | | | | | | |
| Age >40 years |  |  |  | | **0.560 (0.134)** | **<0.001** | **(0.297;0.823)** |
| Time since diagnosis <1 years | *(ref.)* | | | | | | |
| Time since diagnosis 1-5 years |  |  |  | | 0.382 (0.222) | 0.085 | (-0.053;0.817) |
| Time since diagnosis >=6 years |  |  |  | | 0.036 (0.615) | 0.953 | (-1.169;1.242) |
| Age of first symptoms <=20 years | *(ref.)* | | | | | | |
| Age of first symptoms 21-30 years |  |  |  | | -0.35 (0.423) | 0.408 | (-1.178;0.479) |
| Age of first symptoms >30 years |  |  |  | | **-0.946 (0.226)** | **<0.001** | **(-1.390;-0.503)** |
| Intercept |  |  |  | | 0.278 (0.435) | 0.523 | (-0.574;1.13) |
| *Generalized Linear Model* | | | | | | | |
| Age <=40 years | *(ref.)* | | | | | | |
| Age >40 years | **-0.473 (0.230)** | **0.040** | **(-0.924;-0.023)** | | -0.235 (0.218) | 0.279 | (-0.662;0.191) |
| Time since diagnosis <1 years | *(ref.)* | | | | | | |
| Time since diagnosis 1-5 years | -0.449 (0.323) | 0.165 | (-1.082;0.184) | | -0.341 (0.350) | 0.329 | (-1.028;0.345) |
| Time since diagnosis >=6 years | -0.255 (0.555) | 0.646 | (-1.342;0.833) | | -0.648 (0.509) | 0.203 | (-1.646;0.350) |
| Age of first symptoms <=20 years | *(ref.)* | | | | | | |
| Age of first symptoms 21-30 years | -0.394 (0.268) | 0.142 | (-0.920;0.132) | | -0.533 (0.363) | 0.142 | (-1.245;0.179) |
| Age of first symptoms >30 years | -0.252 (0.183) | 0.169 | (-0.612;0.107) | | 0.010 (0.556) | 0.986 | (-1.081;1.100) |
| Intercept | **10.198 (0.309)** | **<0.001** | **(9.592;10.804)** | | **11.091 (0.429)** | **<0.001** | **(10.249;11.932)** |
| Employed (N=131) | | | | | | | |
|  | **Direct health costs** | | | **Direct non health costs** | | | |
|  | **Coef. (Std.Err)** | **P-value** | **(95%CI)** | | **Coef. (Std.Err)** | **P-value** | **(95%CI)** |
| *Probit (Prob. of not incurring in costs)* | | | | | | | |
| Age <=40 years | *(ref.)* | | | | | | |
| Age >40 years | 0.454 (0.395) | 0.251 | (-0.321;1.228) | | 0.358 (0.304) | 0.239 | (-0.238;0.954) |
| Time since diagnosis <1 years | *(ref.)* | | | | | | |
| Time since diagnosis 1-5 years | **-4.385 (0.326)** | **<0.001** | **(-5.024;-3.745)** | | -0.373 (0.228) | 0.102 | (-0.820;0.074) |
| Time since diagnosis >=6 years | **-3.981 (0.192)** | **<0.001** | **(-4.357;-3.606)** | | -0.343 (0.281) | 0.222 | (-0.893;0.208) |
| Age of first symptoms <=20 years | *(ref.)* | | | | | | |
| Age of first symptoms 21-30 years | 0.183 (0.253) | 0.470 | (-0.313;0.678) | | -0.099 (0.152) | 0.516 | (-0.396;0.199) |
| Age of first symptoms >30 years | 0.536 (0.421) | 0.203 | (-0.289;1.362) | | -0.577 (0.348) | 0.097 | (-1.259;0.105) |
| Intercept | **4.815 (0.529)** | **<0.001** | **(3.778;5.852)** | | 0.497 (0.417) | 0.233 | (-0.320;1.315) |
| *Generalized Linear Model* | | | | | | | |
| Age <=40 years | *(ref.)* | | | | | | |
| Age >40 years | 0.128 (0.168) | 0.448 | (-0.202;0.458) | | -0.035 (0.182) | 0.848 | (-0.392;0.322) |
| Time since diagnosis <1 years | *(ref.)* | | | | | | |
| Time since diagnosis 1-5 years | 0.535 (0.274) | 0.051 | (-0.001;1.071) | | 0.203 (0.484) | 0.675 | (-0.745;1.151) |
| Time since diagnosis >=6 years | 0.474 (0.291) | 0.104 | (-0.097;1.045) | | -0.147 (0.381) | 0.700 | (-0.893;0.600) |
| Age of first symptoms <=20 years | *(ref.)* | | | | | | |
| Age of first symptoms 21-30 years | -0.379 (0.209) | 0.070 | (-0.789;0.03) | | -0.025 (0.131) | 0.846 | (-0.282;0.231) |
| Age of first symptoms >30 years | -0.332 (0.193) | 0.086 | (-0.710;0.047) | | -0.288 (0.506) | 0.581 | (-1.271;0.712) |
| Intercept | **8.492 (0.465)** | **<0.001** | **(7.581;9.403)** | | **9.438 (0.351)** | **<0.001** | **(8.749;10.126)** |

^*^For direct health costs only the Generalized Linear Model model is reported for non-workers as just 1 patients resulted in overall costs equal to zero
